# RBFOX1 Regulates Calcium Signaling and Enhances SERCA2 Translation

**DOI:** 10.3390/cells14090664

**Published:** 2025-05-01

**Authors:** Sadiq Umar, Wuqiang Zhu, Fernando Souza-Neto, Ingrid Bender, Steven C. Wu, Chastity L. Healy, Timothy D. O’Connell, Jop H. van Berlo

**Affiliations:** 1 Lillehei Heart Institute, University of Minnesota, Minneapolis, MN 55455, USAzhu.wuqiang@mayo.edu (W.Z.); fsneto@umn.edu (F.S.-N.);; 2 Department of Oral Biology, University of Illinois Chicago, Chicago, IL 60612, USA; 3 Department of Cardiovascular Medicine, Physiology and Biomedical Engineering, Mayo Clinic Arizona, Phoenix, AZ 85059, USA; 4 Department of Integrative Biology and Physiology, University of Minnesota, Minneapolis, MN 55455, USA; 5 Stem Cell Institute, University of Minnesota, Minneapolis, MN 55455, USA

**Keywords:** calcium signaling, heart failure, gene regulation, cardiac hypertrophy

## Abstract

RBFOX1 is an RNA-binding protein that regulates alternative splicing and RNA processing in the neurons, skeletal muscle, and heart. We intended to define the role of RBFOX1 in regulating calcium homeostasis to maintain normal cardiac function. We generated cardiomyocyte-specific *Rbfox1* gene-deletion mice (cKO). The cardiomyocyte-specific deletion of RBFOX1 was confirmed by Western blotting and immunohistochemistry. The cKO mice showed mild hypertrophy and depressed cardiac function under homeostatic conditions, which did not deteriorate with age. Pressure overload by trans-aortic constriction (TAC) caused exaggerated cardiac hypertrophy and accelerated heart failure in cKO compared with wild-type mice. We performed Western blotting to assess the expression of important Ca^2+^-handling proteins, which showed alterations in the phosphorylation of PLN and CAMKII and decreased expression of SERCA2. We measured the Ca^2+^ dynamics and noted significantly delayed Ca^2+^ reuptake into the sarcoplasmic reticulum. Importantly, the decrease in SERCA2 expression was not due to reduced mRNA expression or altered splicing. To assess the possibility of the post-transcriptional regulation of SERCA2 expression by RBFOX1, we performed RNA immunoprecipitation (RIP), which showed the binding of RBFOX1 protein to *Serca2* mRNA, which was confirmed in luciferase assays with the *Serca2a* 3′-untranslated region fused to luciferase. Finally, we performed a puromycin incorporation experiment, which showed that RBFOX1 enhances SERCA2 protein translation. Our results show that RBFOX1 plays a crucial role in regulating the expression of Ca^2+^-handling genes to maintain normal cardiac function. We show an important post-transcriptional role of RBFOX1 in regulating SERCA2 expression.

## 1. Introduction

The role of upstream kinases and transcription factors in regulating the response of the heart to stress have been broadly studied over the past years, which has led to the identification of many regulators of cardiomyocyte hypertrophy that might also affect the propensity to develop heart failure [1]. However, the role of the post-transcriptional processing of RNA in the response of the heart to increased stress is only recently beginning to be explored. One important example of post-transcriptional processing is alternative RNA splicing, which is a complex and finely orchestrated post-transcriptional process to generate multiple functional RNAs and proteins from a single gene [2,3]. The process of splicing is highly conserved during evolution and regulated by various RNA-binding proteins. These RNA-binding proteins (RBPs) provide a robust and versatile mechanism for regulating gene expression and influence pre-mRNA splicing, RNA localization, and stability [3]. RBPs can bind RNA through conserved recognition motifs, and one of the most abundant motifs that regulates alternative splicing is the Rbfox motif [4,5]. Rbfox proteins have a conserved RNA recognition motif (RRM)-type RNA-binding domain that binds the hexanucleotide (U)GCAUG with great specificity [5,6]. The Rbfox family consists of an ancient family of sequence-specific RNA-binding proteins (RBPs) and contains three genes: *Rbfox1*, *Rbfox2*, and *Rbfox3*. This family of RNA-binding proteins plays critical roles in multiple tissues, both during development and in adulthood [7]. *Rbfox1* is selectively expressed in the brain, heart, and skeletal muscle, while *Rbfox2* is widely expressed in most tissues, including the brain, skeletal muscle, and heart, and the expression of *Rbfox3* appears to be restricted to neurons [8,9].

Studies looking into the function of RBFOX1 in the brain and in skeletal muscle have shown that RBFOX1 has an important role in regulating the alternative splicing of genes that are important for Ca^2+^ homeostasis [9,10]. Ca^2+^ is a highly versatile intracellular signal that can regulate many different cellular functions. Abnormalities in Ca^2+^ handling contribute to many different diseases, such as hypertension, heart disease, diabetes, manic depression, and Alzheimer’s disease [11]. Ca^2+^ homeostasis is particularly important in depolarizing cells, such as neurons, skeletal muscle fibers, and cardiac cells, where the removal of Ca^2+^ from the cytoplasm of cells is tightly controlled to maintain the resting level of Ca^2+^. The most important mechanism, in terms of Ca^2+^ throughput, is SERCA. In the heart, SERCA2 primarily controls cytosolic Ca^2+^ removal and determines cardiac relaxation and contraction [12]. SERCA2 is a critical regulator of cardiac contraction, and the reduced expression of SERCA2 is reported to be associated with heart failure in rodent, porcine, and human hearts [13]. Heterozygous mice with a partial loss of *Serca2* expression show significantly reduced cardiomyocyte contractility and sarcoplasmic reticulum (SR) Ca^2+^ load [13]. The reduced expression of SERCA2 sensitized mice to pressure overload-induced heart failure [14]. The most important regulator of SERCA activity in the heart is Phospholamban (PLN), which binds to the cytosolic domain of SERCA2. PLN can be phosphorylated at Ser16 or at Thr17 [13]. When phosphorylated at either or both of these sites, the inhibition of SERCA2 is alleviated, and the Ca^2+^ flux into the SR increases. PLN has been shown to be a major regulator of SERCA2 function in the heart by inhibiting its activity and thereby reducing the influx of Ca^2+^ into the SR [13].

A role of RBFOX1 in the development of heart failure was recently established, as both murine and human failing hearts showed a reduced expression of RBFOX1 [15]. The genetic deletion of *Rbfox1* from cardiomyocytes predisposed hearts to develop heart failure in response to cardiac pressure overload [15]. To begin to explain these results, Gao et al. identified that the MEF2 family of transcription factors are a target of RBFOX1-dependent alternative splicing, which contributes to the phenotype in failing hearts [15]. Likely, the role of RBFOX1 is not limited to the splicing of MEF2, and it is possible that additional functions of RBFOX1 are crucial to maintain normal cardiac function and provide protection against heart failure.

Given the importance of Ca^2+^ homeostasis in cardiac function, we explored a role for RBFOX1 in regulating Ca^2+^ homeostasis as a contributing factor to heart failure. We show that RBFOX1 is important to maintain normal cardiac function and find evidence for a direct interaction between RBFOX1 protein and *Serca2* mRNA, resulting in enhanced translation. These results show that RBFOX1 is a critical regulator of Ca^2+^ homeostasis in the heart.

## 2. Materials and Methods

### 2.1. Animals

LoxP-targeted *Rbfox1* mice were obtained from the Jackson Laboratory (Bar Harbor, ME, USA; Stock # 014089) [10]. We generated cardiomyocyte-specific knockout mice by crossbreeding loxP-targeted *Rbfox1* mice with a cardiomyocyte-specific *Nkx2-5* Cre mouse line [16]. The animals were euthanized at the end of the experiments by isoflurane anesthesia, with the verification of a sufficient plane of anesthesia by toe pinch, followed by cervical dislocation and excision of the heart. All the animal procedures were performed according to the NIH guidelines and approved by the University of Minnesota Institutional Animal Care and Use Committee.

### 2.2. Animal Surgery, Echocardiography, and Histology

All surgeries were performed on 8–12-week-old mice. Both male and female mice were included. Transverse aortic constriction (TAC) was performed as previously described [16]. Briefly, the surgeries were performed under 2% isoflurane anesthesia with long-acting buprenorphine-SR as analgesic (ZooPharm, Laramie, WY, USA; 50 µL sq). After the verification of anesthesia, the mice were intubated and ventilated; a parasternal incision was made, and a blunt 27G needle was ligated onto the transverse aorta between the brachiocephalic and left carotid arteries. After the removal of the needle, the wound was closed, and the mice were allowed to recover. Sham surgeries did not undergo ligation of the transverse aorta. The cardiac function and dimensions were measured by echocardiography using a Vevo2100 instrument (Visualsonics, Toronto, ON, Canada) [17]. The B-mode and 2D M-mode images were obtained at the parasternal long-axis and short-axis views, respectively. The left ventricular (LV) fractional shortening was calculated using LV internal diameters at the end of systole and diastole (LVIDs and LVIDd, respectively) according to the following formula: ([LVIDd − LVIDs]/LVIDd) × 100 (%). Two weeks after TAC, the mice were euthanized, and hearts were harvested for the analysis of heart weight/body weight ratio calculations. For histological analysis, adult hearts were fixed overnight in 10% formalin-containing phosphate-buffered saline and dehydrated for paraffin embedding. Serial 10 µm heart sections were stained with sirius red and fast green to quantify fibrosis. The sections were stained with cleaved caspase 3 (Promega, Madison, WI, USA) and counterstained with nuclear fast red to quantify cardiomyocyte apoptosis. Immunohistochemistry for RBFOX1 was performed on paraffin-embedded sections using antigen-retrieval Citra Plus solution, followed by staining with RBFOX1 (Novus Biologics, Centennial, CO, USA), DESMIN (Millipore, Burlington, MA, USA), and SERCA2 (Thermo, Waltham, MA, USA) and by secondary antibody staining or by wheat germ agglutinin (Vector Laboratories, Newark, CA, USA) mixed with DAPI to counterstain nuclei. The microscopy imaging and analysis were performed at the University Imaging Centers, University of Minnesota, on a Nikon C2 confocal microscope. Heart tissue for electron microscopy was fixed and submitted to the University Imaging Center microscopy core for sectioning and imaging on a Philips CM12 Transmission Electron Microscope.

### 2.3. Western Blotting

The cells or tissue samples were homogenized in lysis buffer containing 50 mM Tris pH 7.6, 150 mM NaCl, 1 mM EDTA, 1% Triton X-100, 0.5% deoxycholate, 0.1% SDS, and a protease/phosphatase inhibitor cocktail. The protein concentration was measured by BCA (Pierce™ BCA Protein Assay Kit, Thermo, Waltham, MA, USA). Equal amounts of protein were loaded on SDS-polyacrylamide gels and separated by electrophoresis and transferred onto nitrocellulose (GE, Amersham, UK). The blots were probed using antibodies specific for p-PLN s16 (EMD-07-052, Millipore, Burlington, MA, USA; 1:1000), p-PLN t17 (Badrilla-A010-13, Leeds, UK; 1:1000), p-TROPONIN I s23/24(CST-4002S, Danvers, MA, USA; 1:1000), p-CAMKII t286 (CST-12716S, Danvers, MA, USA; 1:1000), oxi-CAMKII (EMD-07-1387, Millipore, Burlington, MA, USA; 1:1000) CAMKII (CST-4436S, Danvers, MA, USA; 1:1000), PLN (Sigma-SAB2701037, St. Louis, MO, USA; 1:1000), Troponin I (CST-4002S, Danvers, MA, USA; 1:1000), SERCA2 (Badrilla-A010-23L, Leeds, UK; 1:20,000), NCX (Swant-R3F1, Burgdorf, Switzerland; 1:1000), Calmodulin (Abcam45689, Cambridge, UK), and GAPDH (Fitzgerald Ind. Int.-10R-G109a, Gardner, MA, USA; 1:5000). Antibody-probed blots were developed with Enhanced Chemiluminescence (Pierce, Thermo, Waltham, MA, USA) and visualized with a Bio Rad Chemidoc system (Hercules, CA, USA). The blots were stripped and re-probed with GAPDH to ensure equal protein loading.

### 2.4. Isolation of Adult Cardiomyocytes for Ca^2+^ Measurements

Cardiomyocytes were isolated as previously described [18]. In brief, the heart was rapidly excised from animals that were anesthetized with 2% isoflurane and placed in perfusion buffer containing 120 mM NaCl, 14.7 mM KCl, 0.6 mM KH_2_PO_4_, 0.6 mM Na_2_HPO_4_, 4.6 mM NaHCO_3_, 1.2 mM MgSO_4_, 5.5 mM glucose, 10 mM HEPES, 10 mM 2,3-butanedione monoxime (BDM), and 30 mM taurine (buffer A). The heart was retrograde-perfused with buffer A for 4–5 min, then with buffer A containing 2.4 mg/mL collagenase type II (Worthington Biochemical Corp., Lakewood, NJ, USA) at 37 °C. After 3 min of enzyme perfusion, 40 μM Ca^2+^ was added to the enzyme solution. The heart was perfused for a total of 8–12 min After perfusion, the ventricles were separated from the atria and minced. Myocyte stop buffer was added to inactivate the proteases. Ca^2+^ was gradually added back, after which the myocytes were incubated with 1 μM Fura-2 acetoxymethyl ester (Molecular Probes, Eugene, OR, USA) for 20 min in Tyrode’s buffer (140 mmol/L NaCl, 10 mmol/L glucose, 10 mmol/L HEPES, 4 mmol/L KCl, 1 mmol/L MgCl_2_, pH 7.45) with 1.2 mM Ca^2+^ at room temperature. After being loaded, the myocytes were paced at 1 Hz at 20 V and continuously perfused with Tyrode’s buffer. The calcium dynamics were determined at room temperature using the Fluorescence and Contractility System (IonOptix LLC, Westwood, MA, USA), operating at an emission wavelength of 510 nm with excitation wavelengths of 340 and 380 nm, as published previously [19].

### 2.5. Neonatal Rat Ventricular Cardiomyocyte Isolation

Sprague Dawley rats were purchased from Charles River Laboratories (Wilmington, MA, USA). Neonatal rat pups were euthanized by decapitation on day 1, in accordance with the AVMA guidelines for the euthanasia of animals. Cardiomyocytes were isolated from the newborn pups using a neonatal cardiomyocyte isolation kit (Worthington Biochemical Corp., Lakewood, NJ, USA). Briefly, the hearts were removed from the pups and washed in HBSS and digested with trypsin overnight (16–20 h) at 4 °C followed by a trypsin inhibitor for 15 min and finally digested in collagenase for an hour at 37 °C. The cells were pre-plated to remove fibroblasts, counted, and seeded in gelatin-coated 6-well plates. The cells were transfected with Rbfox1siRNA and Negative siRNA (100 pmol) for 72 h and harvested in a RIPA buffer for Western blotting.

### 2.6. Luciferase Assay

A 778 bp fragment of the 3′UTR of the murine *Serca2a* gene was amplified by polymerase chain reaction (PCR) using forward and reverse primers (forward: 5′-CCGCTTCCTAAACCATTTGCAG and reverse: 5′-TGAGGGTTTATCGTAGAATAGATTTATTTACCTG). PCR was performed using Phusion DNA Polymerase (New England BioLabs Inc., Ipswich, MA, USA) under the conditions of 97 °C for 2 min, 96 °C for 20 s, and 68 °C for 2 min and 30 s, for a total of 35 cycles. The PCR product was subcloned into a pmirGLO Dual-luciferase miRNA expression vector (Promega Corp., Madison, WI, USA). A similar construct, in which all the putative Rbfox1-binding sites were mutated, was purchased from Genewiz (South Plainfield, NJ, USA) and similarly subcloned into pmirGLO. HEK293 cells (ATCC, Manassas, VA, USA) were transfected with Rbfox1 expression vector, *SERCA2a* 3′UTR pmirGLO, and/or mutated *SERCA2a* 3′UTR using Lipofectamine 2000 according to the manufacturer’s instructions. After 24 h, the luciferase activity was detected on a Cytation3 (Biotek Corp., Winooski, VT, USA) plate reader using a Dual-Luciferase Reporter Assay System (Promega Corp., Madison, WI, USA).

### 2.7. RNA-Protein Immunoprecipitation

The immunoprecipitation of RNA complexed with RBFOX1 was performed as described with minor modifications [20]. Briefly, heart tissue from control and knockout mice was homogenized in PBS with 1% Triton X-100; the nuclei were pelleted by centrifugation at 2500× *g* for 15 min. The nuclear pellet was resuspended in 1 mL RIPA buffer (150 mM KCl, 25 mM Tris pH 7.4, 5 mM EDTA, 0.5 mM DTT, 0.5% NP40, protease and phosphatase inhibitors, 100 U/mL SUPERase In). The resuspended nuclei were sonicated followed by pelleting of the nuclear membrane and debris. Rbfox1 antibody (Millipore, Burlington, MA, USA) was added and incubated overnight at 4 °C with gentle rotation. Next, 40 μL of protein A/G beads was added and incubated for 2 h at 4 °C with gentle rotation. The beads were pelleted at 2500 rpm for 30 s; the supernatant was removed, and the beads were triple washed in RIPA buffer, followed by a wash in PBS. RNA was eluted from the beads by resuspension in 1 mL of Trizol, followed by RNA isolation and cDNA synthesis. Quantitative PCR for *Serca2* was performed using the SYBR green dye (Bio-Rad, 172–5124, Hercules, CA, USA) on an ABI-7900 real-time PCR detection system (Applied Biosystems, Waltham, MA, USA). IP against Calmodulin (Abcam, Cambridge, UK) was performed on cardiac lysate.

### 2.8. Assessment of Newly Synthesized Serca2

Cardiomyocytes were isolated from Sprague Dawley newborn pups. The cells were transfected with *Rbfox1* siRNA or control siRNA (100 pmol) for 72 h. Next, the cells were treated with puromycin (1 μM) for 30 min, followed by harvesting in a RIPA buffer for Western blotting and immunoprecipitation. The cell lysates were immunoprecipitated using a specific puromycin antibody. Next, a Western blot was performed for SERCA2.

### 2.9. Statistical Analysis

All the results are presented as means with error bars representing the standard error of the mean. Statistical tests were performed using Prism 10. Student *t* tests were performed to compare means between 2 groups. Comparisons between multiple groups were performed using regular or two-way ANOVA with Dunnett’s post hoc analysis or Tukey’s multiple comparisons test, respectively. A *p* value below 0.05 was considered statistically significant.

## 3. Results

### 3.1. Heart-Specific Rbfox1 Deletion Results in Reduced Cardiac Function and Altered Calcium-Handling-Protein Expression

**Figure 1:**
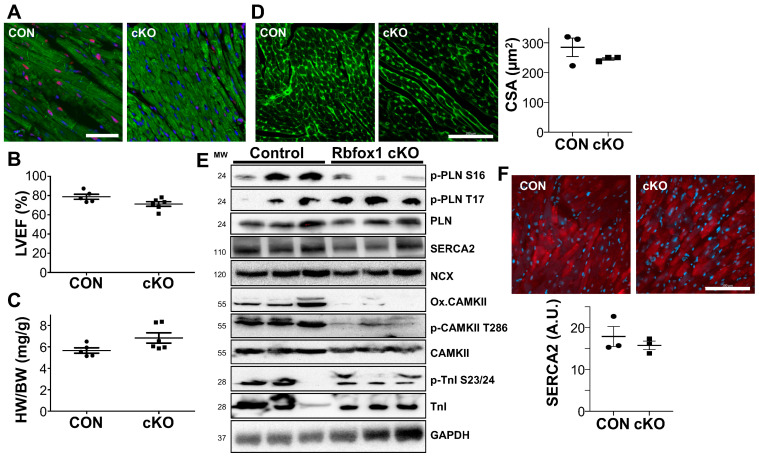
Cardiac specific deletion of Rbfox1 alters calcium handling protein expression. (**A**). Immunostaining of cardiac sections with RBFOX1 (Red) DAPI (blue) and Desmin (Green) from control (CON) and cardiomyocyte-specific *Rbfox1* knock-out mice (cKO), bar = 25 µm (**B**). Echocardiographic assessment of Left Ventricular Ejection Fraction (LVEF) in Control and cKO mice (n = 5 and 6). (**C**). Analysis of heart weight/body weight ratio (HW/BW) in Control and cKO mice (n = 5 and 6). (**D**). Representative staining of wheat-germ agglutinin to measure histological cross-sectional area of cardiomyocytes with quantification, bar = 100 µm (n = 3 and 3) (**E**). Western blot analysis of calcium handling proteins; pospho-PLN (ser16 and thr17), PLN, SERCA2, NCX, oxidized-CAMKII, phospho-CAMKII (thr286), CAMKII, phospho-Troponin I (ser23/24), and Troponin I in heart lysates of 1-month old mice. GAPDH was used as a loading control. (**F**). Representative immunostaining for SERCA2 (red) and DAPI (blue) with quantification of SERCA2 intensity, bar = 100 µm (n = 3 and 3).

We generated cardiomyocyte-specific *Rbfox1* gene-deletion mice by crossbreeding *Rbfox1* loxP-targeted mice with *Nkx2-5* Cre mice [10]. We confirmed the deletion of RBFOX1 by immunohistochemistry for RBFOX1, in which we observed mainly nuclear staining in cardiomyocytes in the control mice (Ctrl), which was absent in RBFOX1-deleted mice (cKO) (Figure 1A). Since it was previously shown that RBFOX1 deletion results in mild cardiac dysfunction in young adult mice, we wanted to assess whether cardiac dysfunction and/or cardiac hypertrophy were already present at a young age [15]. To that effect, we measured the cardiac function by echocardiography at 1 month of age and noted a small, but not significant, reduction in the ejection fraction (Figure 1B and Supplemental Table S1). At that age, there was no change in cardiac hypertrophy, which was independently confirmed by immunohistochemistry for the cross-sectional area (CSA) of cardiomyocytes (Figure 1C,D and Supplemental Table S2). To assess a potential role for RBFOX1 in regulating Ca^2+^ homeostasis, we performed Western blotting for many of the central Ca^2+^ regulators. The most notable differences included enhanced phosphorylation of Phospholamban at serine 17 but reduced phosphorylation of Calmodulin-dependent Kinase II at Threonine 286, while SERCA2 showed a non-significant reduction in expression (Figure 1E and Supplemental Figure S1A–G). We performed immunohistochemical staining for SERCA2, which showed no difference in the abundance of SERCA2 protein (Figure 1F). These results suggest that RBFOX1 is dispensable for cardiac development, and mice develop normally when *Rbfox1* is deleted from cardiomyocytes.

**Figure 2:**
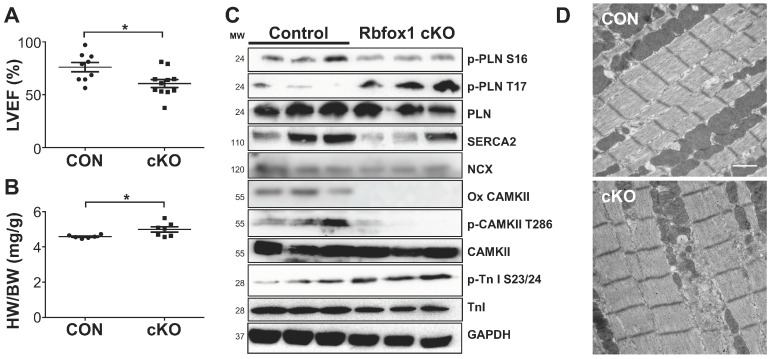
Cardiac specific deletion of *Rbfox1* alters calcium handling protein expression and causes mild cardiac dysfunction. (**A**). Echocardiographic assessment of Left Ventricular Ejection Fraction (LVEF) in Control and cKO mice (n = 9 and 11). (**B**). Analysis of heart weight/body weight ratio (HW/BW) in Control and cKO mice (n = 6 and 7). (**C**). Western blot analysis of calcium handling proteins; p-PLN (ser16 and thr17), PLN, SERCA2, NCX, oxi-CAMKII, p-CAMKII (thr286), CAMKII, p-Troponin I (ser23/24), and Troponin I in heart lysates of 1-month old mice. GAPDH was used as a loading control. (**D**). Electron microscopy of control and cKO mouse hearts. Bar = 1 µm. * *p* < 0.05 vs. Control.

Next, we again assessed cardiac function and hypertrophy, but now at 3 months of age. At this age, we observed a mild but significant reduction in cardiac function, as well as mild but significant cardiac hypertrophy (Figure 2A,B and Supplemental Tables S1 and S2). We again performed Western blotting for many of the central Ca^2+^ regulators, and again noticed enhanced phosphorylation of PLN at T17, as well as reduced phosphorylation of CAMKII at T286, now accompanied by a significant reduction in SERCA2 expression (Figure 2C and Supplemental Figure S2A–G). These changes are consistent with the reduced activation of the major Ca^2+^ pump SERCA2. Importantly, the observed reduction in SERCA2 expression in cKO mice was not accompanied by alterations in NCX expression. While the phosphorylation of sarcomeric Troponin I was not dramatically altered, we did note the reduced phosphorylation and oxidation of CAMKII, which could suggest an intrinsic defect in CAMKII activation. To assess this possibility, we performed immunoprecipitation against Calmodulin, followed by Western blotting for CAMKII, which showed no difference between control and cKO mice (Supplemental Figure S2H). We also did not find major abnormalities at the ultrastructural level using electron microscopy (Figure 2D).

**Figure 3:**
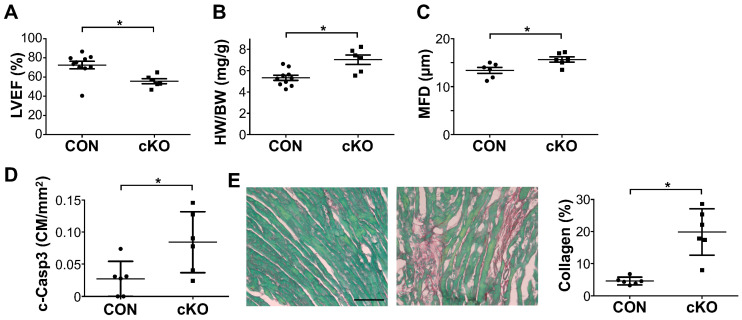
*Rbfox1* cKO mice display mild cardiac dysfunction and apoptosis with aging. (**A**). Echocardiographic assessment of left ventricular ejection fraction (LVEF) in Control and cKO mice at 12 month of age (n = 10 and 6). (**B**). Analysis of heart weight/body weight ratio in Control and cKO mice at 12 months of age (n = 10 and 6). (**C**). Analysis of minimum fiber diameter (MFD) of cardiomyocytes from histological sections (n = 6 and 6). (**D**). Quantification of cleaved caspase-3 staining within cardiomyocytes, indicative of cardiomyocyte apoptosis (n = 6 and 6). (**E**). Above: Representative images of fast green, Sirius red staining for collagen deposition in Control and cKO mice (bar = 100 µm). Below: Quantification of collagen in control and cKO mice (n = 6 and 6) * *p* < 0.05 vs. Control.

Since we noted a small but significantly reduced cardiac function, we wanted to assess whether this would deteriorate with age and cause heart failure. We assessed cardiac function by echocardiography in aged mice at 12 months of age, which showed a similar level of cardiac dysfunction as 3-month-old mice, indicating stable but not progressive cardiac dysfunction (Figure 3A). We again measured cardiac hypertrophy, assessed by the heart-to-body weight ratio, which was significantly higher in cKO mice (Figure 3B). We further confirmed that this was due to cardiomyocyte hypertrophy by measuring the minimum fiber diameter of cardiomyocytes from histological sections (Figure 3C) [21]. Furthermore, we measured significantly higher levels of cleaved CASPASE 3, indicative of cardiomyocyte apoptosis, as well as increased collagen deposition (Figure 3D,E).

### 3.2. Rbofx1 Is Increased in Pressure Overload Stimulation and Protects the Heart

**Figure 4:**
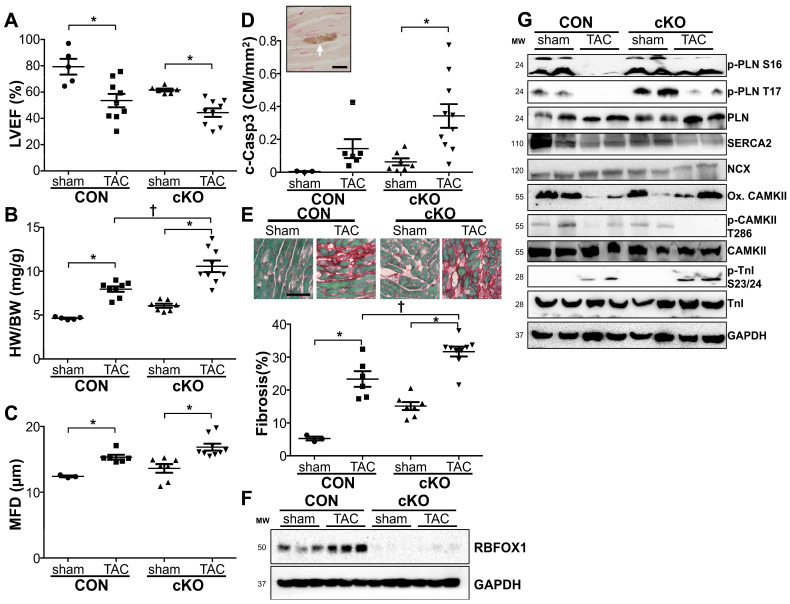
*Rbfox1* cKO mice are sensitive to TAC-induced cardiac dysfunction and show dysregulation of calcium handling proteins. (**A**). Echocardiographic assessment of left ventricular ejection fraction (LVEF) in Control and cKO mice 2 weeks after sham or TAC surgery (n = 5, 9, 6, 9). (**B**). Analysis of heart weight/body weight ratio in Control and cKO mice 2 weeks after sham or TAC surgery (n = 5, 8, 7, 9). (**C**). Minimal fiber diameter of cardiomyocytes from histological sections of control and cKO mice after sham or TAC surgery (n = 3, 6, 7, 9). (**D**). Quantification of cardiomyocyte cleaved caspase-3 staining in Control and cKO mice 2 weeks after sham or TAC surgery. Image shows example of cardiomyocyte that is positive for cleaved caspase-3 staining (white arrow, bar = 10 µm). (**E**). Fast green, Sirius red staining for collagen deposition in Control and cKO mice 2 weeks after sham or TAC surgery. Images show representative examples (bar = 100 µm). (**F**). Western blot analysis of Rbfox1 in Control and cKO mice 2 weeks after sham or TAC surgery. (**G**). Western blot analysis of calcium handling proteins; p-PLN (ser16 and thr17), PLN, SERCA2, NCX, oxi-CAMKII, p-CAMKII (thr286), CAMKII, p-Troponin I (ser23/24), and Troponin I in heart lysates of Control and cKO mice 2 weeks after sham or TAC surgery. GAPDH was used as a loading control. * *p* < 0.05 vs. sham † *p* < 0.05 vs. Control TAC.

RBFOX1 was previously shown to regulate the response to cardiac pressure overload stimulation [15]. We confirmed that, indeed, RBFOX1 is a critical regulator of the response of the heart to pressure overload stimulation. We performed transverse aortic constriction (TAC) in adult mice to induce cardiac pressure overload. This resulted in depressed cardiac function with an increase in cardiac hypertrophy as expected in control mice. The depressed cardiac function and cardiac hypertrophy observed in cKO mice at baseline was further exaggerated in response to TAC (Figure 4A–C and Supplemental Figure S3A and Supplemental Tables S1 and S2). We measured significantly higher levels of cardiomyocyte apoptosis in cKO mice in response to TAC than those in control mice in response to TAC (Figure 4D). Furthermore, collagen deposition in response to pressure overload was also significantly enhanced in cKO mice (Figure 4E). These results show an important role for RBFOX1 in protecting the heart against pressure overload-induced cardiac dysfunction and its sequelae. To begin to explore how RBFOX1 may regulate processes in the heart in response to pressure overload, we first measured RBFOX1 protein expression in sham or TAC-operated animals, which showed enhanced expression in control mice, while cKO mice had undetectable levels of RBFOX1 (Figure 4F). We next explored the effects of pressure overload on the major regulators of Ca^2+^ homeostasis (Figure 4G and Supplemental Figure S3B–H). As expected, we noted alterations in the activation or expression of regulators of Ca^2+^ homeostasis in control mice in response to TAC. When compared with cKO mice after TAC, we again noted a decrease in phosphorylated CAMKII, although this did not reach statistical significance. Furthermore, although the phosphorylation of Phospholamban was reduced in both control and cKO mice in response to TAC, the cKO mice maintained a significantly higher level of PLN phosphorylation at T17 after TAC than the control mice. Troponin I phosphorylation was dramatically enhanced to stimulate contraction but did not show differences in response to TAC between control and cKO mice. Arguably, the most important finding in terms of changes in Ca^2+^ regulators was a decrease in the overall SERCA2 protein expression levels (Figure 4G and Supplemental Figure S3B,H).

### 3.3. Rbfox1 Regulates Calcium Dynamics

**Figure 5:**
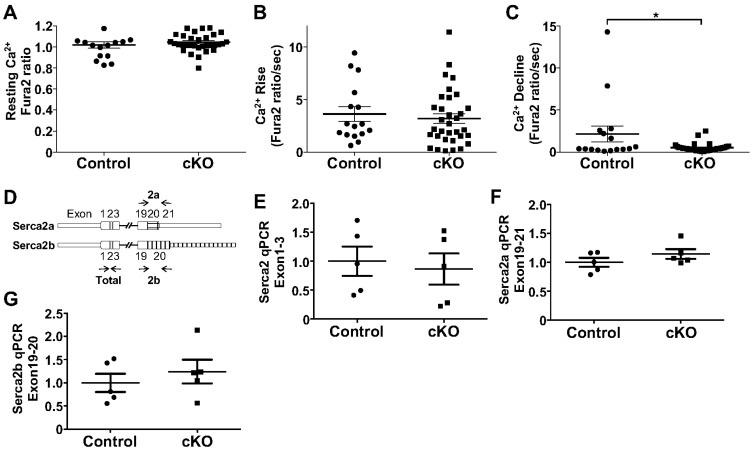
RBOFX1 regulates calcium dynamics. (**A**). Resting Ca^2+^ level measured by Fura2 ratio (340/380) in cardiomyocytes isolated from Control and cKO mice (n = 3 and 4 mice). (**B**). Rise in Ca^2+^ level upon paced transient measured by Fura2 ratio (340/380) in cardiomyocytes isolated from Control and cKO mice (n = 3 and 4 mice). (**C**). Decline Ca^2+^ level measured by Fura2 ratio (340/380) in cardiomyocytes isolated from Control and cKO mice (n = 3 and 4 mice). (**D**). Design of PCR primers to detect overall Serca2 mRNA levels (exon 1-3) or *Serca2a* vs. *Serca2b* specific isoforms. (**E**). qPCR for total Serca2 mRNA expression from Control and cKO hearts at 3 months of age (n = 5 and 5). (**F**). qPCR for total *Serca2a* mRNA expression from Control and cKO hearts at 3 months of age (n = 5 and 5). (**G**). qPCR for total *Serca2b* mRNA expression from Control and cKO hearts at 3 months of age (n = 5 and 5). * *p* < 0.05 vs. Control.

To assess whether the observed changes in SERCA2 protein abundance and other Ca^2+^ regulatory proteins had any consequence on Ca^2+^ homeostasis and cycling, we isolated adult cardiomyocytes from control and cKO mice, loaded them with Fura-2, and measured the Ca^2+^ dynamics (Figure 5A–C). These studies showed no difference in resting Ca^2+^ levels or the Ca^2+^ rise in paced transients but showed a significantly reduced decline, indicating slower Ca^2+^ reuptake, which is consistent with reduced SERCA2 expression and function (Figure 5A–C). Given the well-known role for RBFOX1 in regulating alternative splicing, we first assessed whether RBFOX1 regulated the splicing of *Serca2*. The *Serca2* gene has two splice isoforms that differ in their C-terminal sequence, called *Serca2a* and *Serca2b* (Figure 5D) [22]. Adult hearts mainly express *Serca2a*. Although Western blotting showed downregulation of SERCA2 protein expression in cKO mice, we did not detect the downregulation of *Serca2* mRNA expression using primers that do not distinguish between *Serca2a* and *Serca2b* (Figure 5E). Moreover, when we analyzed the expression of isoform *Serca2a* or *Serca2b* using isoform-specific primers, we did not detect differences between control and cKO mice (Figure 5F,G). These results suggested a possible post-transcriptional role for RBFOX1 in regulating SERCA2 protein abundance.

### 3.4. RBFOX1 Regulates Serca2 Translation

**Figure 6:**
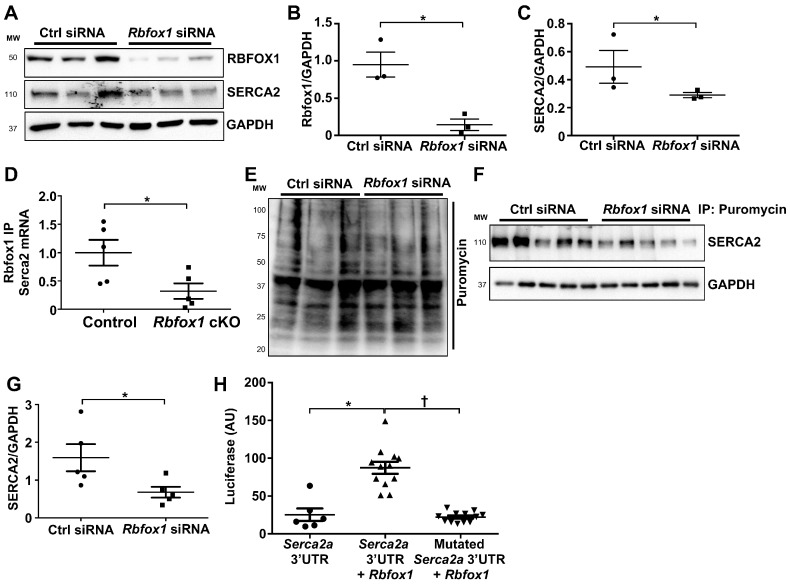
RBFOX1 regulates *Serca2* mRNA translation. (**A**). Western blot analysis of RBFOX1, SERCA2 and GAPDH protein in neonatal rat ventricular cardiomyocytes after transfection with *Rbfox1* or control siRNA. (**B**). Densitometry analysis of Rbfox1 expression corrected to GAPDH after control or *Rbfox1* siRNA transfection (n = 3 and 3). (**C**). Densitometry analysis of SERCA2 expression after control or *Rbfox1* siRNA transfection (n = 3 and 3). (**D**). qPCR expression of *Serca2* mRNA after RNA immunoprecipitation using RBFOX1 antibody in heart lysates of Control and *Rbfox1* cKO mice (n = 5 and 5). (**E**). Western blot for incorporated puromycin in proteins extracted from neonatal rat ventricular cardiomyocytes after transfection with *Rbfox1* or control siRNA. (**F**). Western blot for SERCA2 from protein lysates after immunoprecipitation using puromycin antibody. (**G**). Densitometry analysis of newly formed SERCA2 protein based on puromycin immunoprecipitation in panel (**F**). (n = 5 and 5). (**H**). Dual luciferase assay for Luciferase cDNA plasmid with *Serca2a* 3′UTR or mutated *Serca2* 3′UTR, where RBFOX1 binding sites have been mutated, with or without *Rbfox1* expression vector co-transfection (n = 6, 12 and 12). * *p* < 0.05 vs. Control † *p* < 0.05 vs. *Serca2a* 3′UTR with *Rbfox1*.

To determine whether RBFOX1 could directly regulate *Serca2* mRNA translation, we used rat neonatal cardiomyocytes and transfected these with siRNA against *Rbfox1*. Two days after the transfection of siRNA, we noticed a clear downregulation of RBFOX1, as expected, and also noticed a significant reduction in SERCA2 protein abundance compared with control siRNA (Figure 6A–C). These results potentially suggested that RBFOX1 could directly regulate SERCA2 protein expression. Since *Serca2* mRNA expression was unaltered (Figure 5E–G), we assessed a potential post-transcriptional mode of regulation. First, we assessed whether the RBFOX1 protein could interact with *Serca2* mRNA. We performed immunoprecipitation against RBFOX1 protein and measured *Serca2* mRNA expression in the pull-down in either control or cKO mice, which showed interaction between RBFOX1 and *Serca2* mRNA in control mice but significantly reduced interaction in cKO mice (Figure 6D). These results suggested that RBFOX1 could directly interact with *Serca2* mRNA to regulate its expression. Since the splicing of the *Serca2* gene was unaltered, we assessed whether RBFOX1 could regulate *Serca2* mRNA translation. To that end, we again used rat neonatal cardiomyocytes and treated these with control or *Rbfox1* siRNA. Next, we treated both sets of cardiomyocytes with puromycin, which is incorporated into newly synthesized proteins [23]. We first ran a Western blot for overall puromycin incorporation, which did not show major deficiencies in protein translation (Figure 6E). We then performed immunoprecipitations using an antibody against puromycin and performed Western blotting for SERCA2, which showed a significant reduction in newly synthesized SERCA2 when *Rbfox1* was knocked down (Figure 6F,G). Finally, to determine the precise mechanism of RBFOX1-mediated *Serca2* mRNA translation, we generated luciferase constructs in which either a wild-type *Serca2a* 3′UTR or a mutated *Serca2a* 3′UTR with all the putative RBFOX1-binding sites mutated was inserted after a luciferase complementary DNA. These luciferase constructs were used in a transfection assay in conjunction with *Rbfox1* transfection. Notably, we found that RBFOX1, indeed, enhances luciferase activity in the presence of *Serca2a* 3′UTR, which was significantly blunted when the putative RBFOX1-binding sites in the *Serca2a* 3′UTR were mutated, showing that binding of RBFOX1 to the *Serca2a* 3′UTR enhances mRNA translation (Figure 6H).

## 4. Discussion

The role of RNA-binding proteins in the heart is only beginning to be explored [2,3]. It was recently established that alternative splicing by the RNA-binding protein RBFOX1 is critical to maintain normal cardiac function in zebrafish and to protect the murine heart from pressure overload-induced heart failure [15,24]. The main mechanistic evidence provided was through the alternative splicing of an exon in the important transcription factor *Mef2* [15]. The identification and characterization of other substrates will help to determine the full function of RBFOX1 in the heart. In the brain and skeletal muscles, it is well established that RBFOX1 regulates genes that are important for Ca^2+^ homeostasis [9,10]. For example, *Rbfox1* deletion from skeletal muscle fibers disrupted the ultrastructural integrity of the skeletal muscle fibers with the mislocalization of SERCA1 and RYR1 in muscle fibers resulting in altered Ca^2+^ handling [9]. These changes resulted in significantly impaired muscle function. Although the molecular mechanisms via which *Rbfox1* deletion caused these alterations in SERCA1 and RYR1 localization and function were not explored, it clearly indicated that RBFOX1 is critical for normal Ca^2+^ handling in depolarizing cells, such as neurons and skeletal muscle fibers. We also noted some differences with a prior publication on the role of RBFOX1 in cardiac pressure overload [15]. We observed increased expression of RBFOX1 at 2 weeks after cardiac pressure overload (Figure 4F), a phase in which most mice display compensated hypertrophy, while a prior publication showed reduced RBFOX1 expression at 8 weeks after cardiac pressure overload, when mice have transitioned to the development of heart failure. These differences potentially suggest an important role for RBFOX1 at all stages of the response to cardiac pressure overload, where upregulation is likely important to ensure a compensated response to pressure overload while the later decline could accelerate the development of heart failure.

Since we detected mild cardiac dysfunction already at baseline, we reasoned Rbfox1 must regulate a critical process within the heart. Therefore, we explored the role of Rbfox1 in regulating Ca^2+^ homeostasis and cycling in cardiomyocytes as a mechanism to maintain normal cardiac function and protect the heart from pressure overload-induced hypertrophy and failure. We noticed extensive changes in proteins that are critical for Ca^2+^ homeostasis under baseline conditions. Notably, we observed decreased phosphorylation and oxidization of CAMKII as well as altered phosphorylation of Phospholamban. Combined with the reduced expression of SERCA2, this should result in slower Ca^2+^ reuptake, which is indeed what we observed. An important consequence of slower Ca^2+^ reuptake would be increased Ca^2+^ during diastole, where it might lead to the activation of pathways that can lead to cardiac hypertrophy. Moreover, human cardiac samples from heart failure patients show increased diastolic Ca^2+^. The role of Ca^2+^ in driving cardiac hypertrophy has been extensively studied, and although the precise regulation of local Ca^2+^ remains enigmatic, the overall conclusion from these studies is that increased Ca^2+^ can lead to cardiac hypertrophy and failure [25]. Based on these findings, a therapeutic strategy to increase SERCA2 expression in myocytes was devised [26]. Although animal experiments and an earlier clinical trial showed great promise for this therapeutic strategy, unfortunately the phase IIb clinical trial did not improve patient outcomes [27]. Nevertheless, it is clear that increased Ca^2+^ can result in the further deterioration of cardiac pathology [28]. Here, we showed that genetic deletion of *Rbfox1* causes reduced protein production of SERCA2, leading to impaired Ca^2+^ reuptake by the SR. We furthermore showed that the deletion of *Rbfox1* drives cardiac hypertrophy and cardiac dysfunction in response to pressure overload stimulation.

Furthermore, our results point to an important role for RBFOX1 in regulating mRNA translation through direct interaction with the 3′UTR of *Serca2a*. This role is distinct from the regulation of alternative splicing and adds to the function of RBFOX1 in the heart as an RNA-binding protein. Such a role for RBFOX1 in addition to regulating alternative splicing was recently discovered in the brain. In neurons, it was shown that RBFOX1 can regulate mRNA stability through a conserved RBFOX1 site in 3′-untranslated region (UTR), such that the abundance of transcripts with UGCAUG motifs correlates positively with RBFOX1 expression and knockdown decreases the abundance of these mRNAs, supporting the hypothesis that RBFOX1 enhances mRNA stability [29]. Furthermore, a specific cytoplasmic role for RBFOX1 was identified to regulate the stability and translation of its target mRNAs in the brain [30]. This study proposed two possible mechanisms via which RBFOX1 might regulate expression by binding to the 3′UTR: by increasing mRNA stability and translational efficiency or by interfering with miRNA binding sites. Moreover, the regulation of expression appeared to be dependent on direct binding of RBFOX1 through consensus sequences. This was convincingly shown by generating luciferase constructs that contain the 3′UTR of RBFOX1-regulated genes and then mutating all the potential binding sites. The mutated constructs no longer showed upregulation due to RBFOX1 expression. Here, we showed direct interaction between RBFOX1 and *Serca2* mRNA. Our analysis of the 3′UTR of *Serca2a* indicates it contains at least five RBFOX1 consensus binding elements. Although we did not explore the consequence of mutating all the potential binding sites individually on the expression of a luciferase construct, we convincingly showed that co-expression with RBFOX1 enhanced the expression of luciferase. This shows that RBFOX1 can directly regulate the expression of *Serca2* and potentially other genes that are critical for Ca^2+^ homeostasis, without altering alternative splicing.

It was surprising to observe reduced CAMKII phosphorylation in the cKO mice as well as in the control mice after cardiac pressure overload. Especially if diastolic Ca^2+^ is increased due to reduced SERCA2 expression and activity, we expected this to lead to enhanced CAMKII phosphorylation and activation due to increased activation by Ca^2+^-bound Calmodulin. How the phosphorylation of CAMKII can be reduced in the presence of increased Ca^2+^ remains unknown. It could be that endogenous inhibitors of CAMKII are blocking the activation of CAMKII to counteract the reduced SERCA2 activity or that the increased Ca^2+^ is localized to a different microdomain [31]. However, our assessment for an intrinsic defect in Calmodulin binding to CAMKII did not show differences between control and cKO samples. Perhaps the timing of when we stopped the experiments, which is during the compensated hypertrophy phase, might be a factor in the discrepancy. Another important mechanism through which Ca^2+^ can induce cardiac hypertrophy is through the activation of calcineurin [32]. This is a critical phosphatase that activates NFAT translocation to the nucleus, where it can induce gene transcription. To what extent the Calcineurin/NFAT pathway is activated in absence of RBFOX1 is not clear. Regardless, these are mechanisms that are downstream of the primary mechanism that we have shown here, i.e., the reduced expression of SERCA2 due to the absence of RBFOX1.

A potential limitation of our study is that we only focused on RBFOX1 binding to and regulating *Serca2* mRNA. It is not clear whether the cytoplasmic role of RBFOX1 to regulate and enhance mRNA stability and translation is more widespread than *Serca2*. In the future, we will explore whether RBFOX1 can directly regulate other mRNAs involved in Ca^2+^ homeostasis, such as CAMKII. Furthermore, our Western blotting might give the impression that SERCA2 is almost absent, which is likely not the case and probably a consequence of loading minimal amounts of protein to be able to detect differences in expression. Immunohistochemical staining for SERCA2 showed abundant expression, as is expected. Clearly, SERCA2 is required for proper contraction and relaxation, and the *Rbfox1*-deleted mice lived a relatively normal life up until 12 months of age, which is indicative of no major deficiencies in overall cardiac function.

The fact that RBFOX1 not only has a role in alternative splicing but also regulates translational efficiency shows the complexity of the post-transcriptional process. Although miRNAs have been extensively studied for their role in the development of cardiac hypertrophy and failure, in-depth studies of additional regulators of RNA processing are relatively scarce [33]. This area of research is especially important since it is well known that the correlation between mRNA and protein expression is relatively poor [34]. This can have many potential explanations, such as the activity of miRNAs that can regulate mRNA degradation [35]. However, additional post-transcriptional regulatory mechanisms are most certainly involved. The most important factor that regulates protein abundance is the half-life of proteins. This is likely regulated at the post-translational level due to proteasomal degradation, autophagy, or other means of recycling proteins. However, the process from transcription to translation also has many options for regulation. For example, cells have the ability to guide mRNA molecules to specific sites within the cell to localize the site of translation close to the site at which proteins will be used, such as mitochondria [36,37]. Furthermore, some cells can temporarily ‘store’ mRNA without initiating translation through binding with specific translational initiation factors [38]. In addition, mechanisms that regulate the stability of mRNA and efficiency of mRNA translation are important to fine-tune the cellular machinery to produce the proper proteins at the proper place at the proper time [3]. Many other mechanisms likely exist that further fine-tune the translation of mRNA. For example, a recent study showed dynamic 3′-end formation in mRNA extracted from patients with heart failure, suggesting more widespread regulation of mRNA expression and dynamics underlying disease progression [39]. Whether any of these processes are important in protecting the heart from failing is not well studied.

## 5. Conclusions

In conclusion, we show that RBFOX1 is important for the normal expression of SERCA2 protein production. Deletion of *Rbfox1* from the heart results in reduced SERCA2 expression, with delayed Ca^2+^ reuptake by the SR, giving rise to cardiac dysfunction and hypertrophy. These findings add to the role of RBFOX1 in protecting the heart from stress and add to the recent findings that RBFOX1 regulates *Mef2* alternative splicing. They, furthermore, point to a critical role for RBFOX1 in protecting the heart from failure and might provide a novel therapeutic target for heart failure.

## Data Availability

Data supporting the reported results can be obtained by contacting the corresponding author.

## Supplementary Materials

The following supporting information can be downloaded at https://www.mdpi.com/article/10.3390/cells14090664/s1: Figure S1: Quantification and replicates of Western blot experiments performed at 1 month of age; Figure S2: Quantification and replicates of Western blot experiments performed at 3 months of age; Figure S3: Cell size and protein changes after TAC; Table S1: Overview of echocardiographic measurements; Table S2: Overview of morphometric measurements.

## Abbreviations

The following abbreviations are used in this manuscript:

cKO	Cardiac-specific knockout for Rbfox1
SR	Sarcoplasmic reticulum
TAC	Transverse aortic constriction
