# Supplementary material for: RBFOX1 Regulates Calcium Signaling and Enhances SERCA2 Translation
Source: Cells. 2025 May 1;14(9):664. doi: 10.3390/cells14090664 (PMC12072054; doi:10.3390/cells14090664)
Supplement: Supplementary file 1 [file cells-14-00664-s001.zip › cells-3545102-supplementary.pdf]

Supplemental Material

Rbfox1 Regulates Calcium Signaling and Enhances SERCA2 Translation

Sadiq Umar *, Wuqiang Zhu*, Fernando Souza-Neto, Ingrid Bender, Steven C. Wu, Chastity L. Healy, Timothy D. O'Connell, Jop H. van Berlo

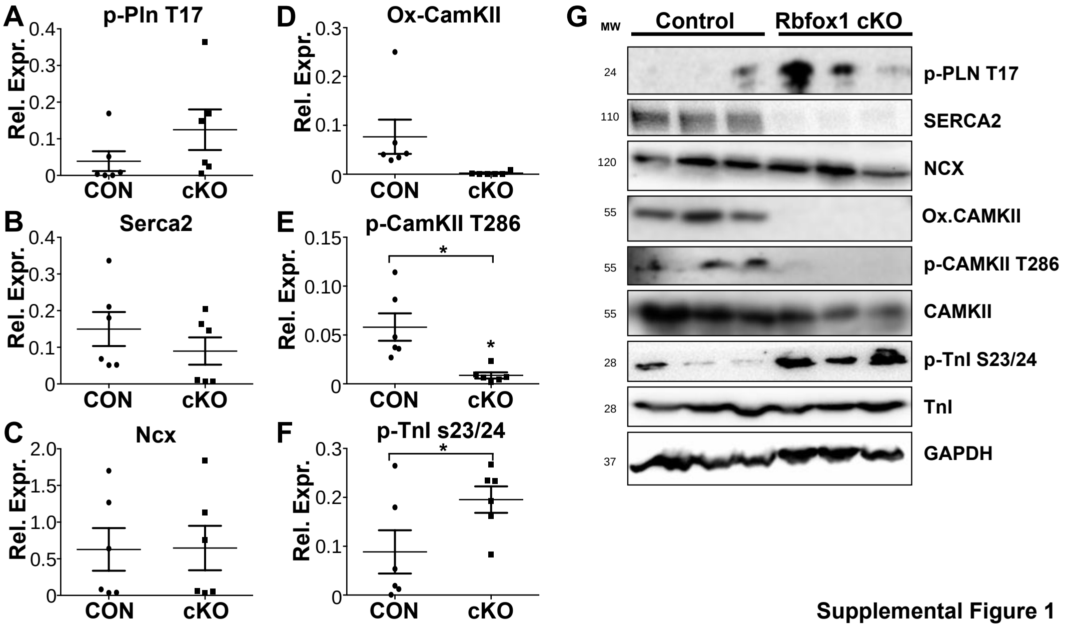

Supplemental Figure 1

Figure S1. Quantification of western blot experiments performed at 1 month of age.
A. Quantification of western blot analysis of phosphorylation of PLN at T17. **B.** Quantification of western blot analysis of SERCA2. **C.** Quantification of western blot analysis of NCX. **D.** Quantification of western blot analysis of oxidized CamKII. **E.** Quantification of western blot analysis of phosphorylation of CamKII at T286. **F.** Quantification of western blot analysis of phosphorylation of TnI at S23/24. All western blots were corrected to GAPDH expression. N=6 for each analysis. *p<0.05 vs Control. **G.** Western blot analysis of indicated proteins in cardiac lysates from control and Rbfox1 cKO mice at 1 month of age.

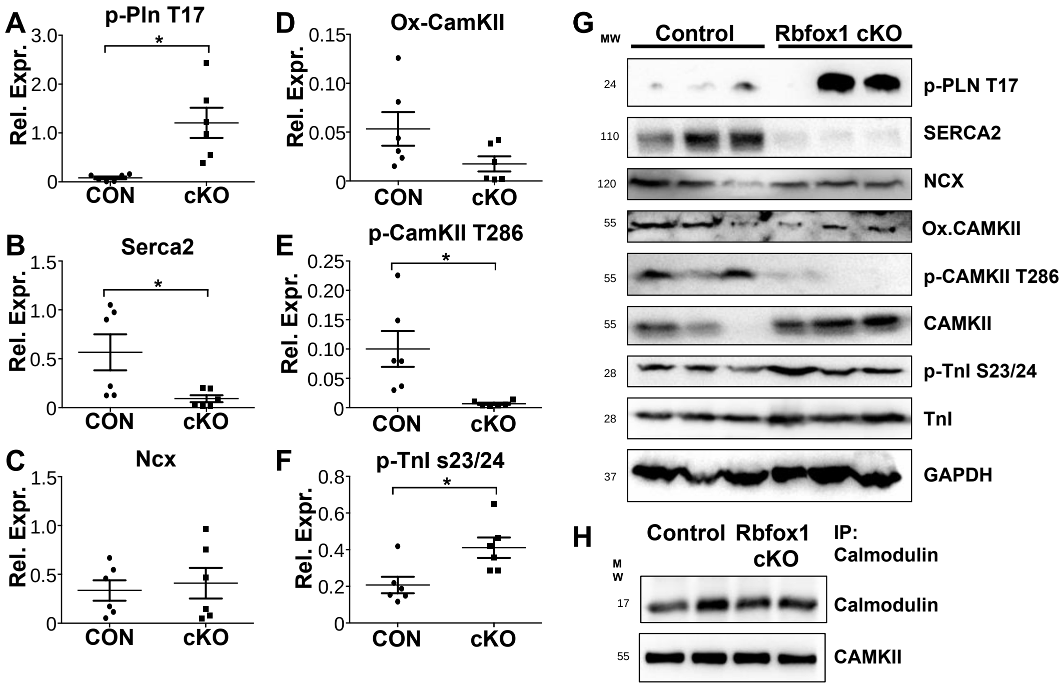

Supplemental Figure 2

Figure S2. Quantification of western blot experiments performed at 3 months of age.

A. Quantification of western blot analysis of phosphorylation of PLN at T17. **B.** Quantification of western blot analysis of SERCA2. **C.** Quantification of western blot analysis of NCX. **D.** Quantification of western blot analysis of oxidized CamKII. **E.** Quantification of western blot analysis of phosphorylation of CamKII at T286. **F.** Quantification of western blot analysis of phosphorylation of TnI at S23/24. All western blots were corrected to GAPDH expression. N=6 for each analysis. *$p<0.05$ vs Control. **G.** Western blot analysis of indicated proteins in cardiac lysates from control and Rbfox1 cKO mice. **H.** Immunoprecipitation of Calmodulin followed by western blotting for Calmodulin and CamKII from cardiac lysate of Control and Rbfox1 cKO mice at 3 months of age.

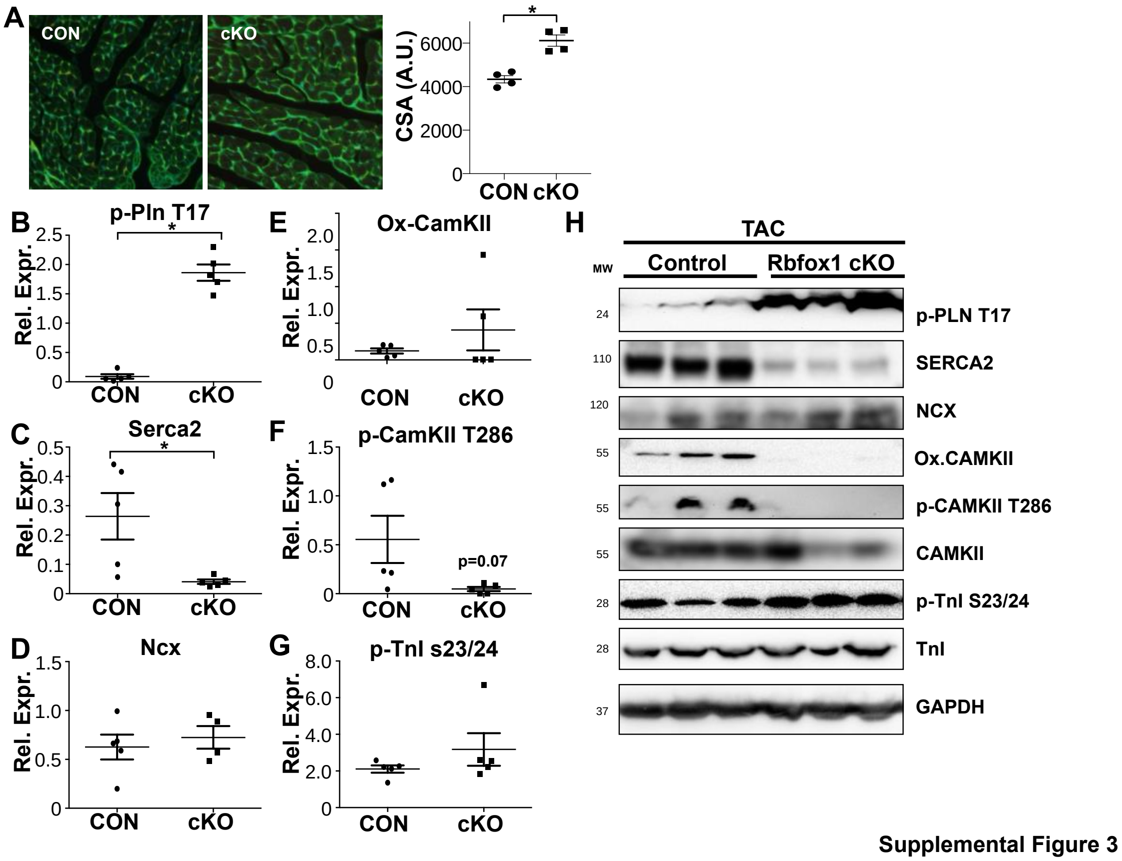

Figure S3. Cell size and protein changes after TAC.

A. Representative immunohistochemistry for Wheat Germ Agglutinin to measure cross-sectional area (CSA) of cardiomyocytes after TAC with quantification to the right. N=4 each, *p<0.01 vs Control. **B.** Quantification of western blot analysis of phosphorylation of PLN at T17. **C.** Quantification of western blot analysis of SERCA2. **D.** Quantification of western blot analysis of NCX. **E.** Quantification of western blot analysis of oxidized CamKII. **F.** Quantification of western blot analysis of phosphorylation of CamKII at T286. **G.** Quantification of western blot analysis of phosphorylation of TnI at S23/24. All western blots were corrected to GAPDH expression. N=4-5 for each analysis. *p<0.05 vs Control. **H.** Western blot analysis of indicated proteins in cardiac lysates from control and Rbfox1 cKO mice after TAC.

Experiment	Genotype	n	HR	LVd	LVs	EF	FS
**1 month**	Wt	5	470±25	3.18±0.34	1.71±0.35	78.8±5.9	46.6±5.6
	cKO	6	463±32	3.42±0.34	2.07±0.34	71.2±6.0	39.8±4.5
**3 months**	Wt	9	489±54	3.38±0.48	1.80±0.60	76.4±13.5	48±12.6
	cKO	11	457±61	3.63±0.51	2.48±0.64*	60.5±12.4*	32.5±9.2*
**TAC**	Wt, sham	5	477±46	3.19±0.40	1.64±0.62	79.3±13.4	50.0±15.8
	Wt, TAC	9	560±84	3.61±0.54	2.63±0.64*	53.4±15.3*	27.7±9.9*
	cKO, sham	7	548±102	3.52±0.56	2.41±0.46	61.1±5.2	32.0±3.3
	cKO, TAC	9	536±29	3.75±0.44	2.94±0.47	44.4±9.8	21.7±5.5

Table S1. Overview of echocardiographic measurements. Shown are averages±SEM. Abbreviations: n, number; HR, heart rate (beats per minute); LVd, Left ventricular dimension during diastole (mm); LVs, Left ventricular dimension during systole (mm); EF, ejection fraction (%); FS, fractional shortening (%); wt, wild type; cKO, cardiac specific Rbfox1 knock-out; TAC, transverse aortic constriction. *p<0.05 vs Wt or sham

Experiment	Genotype	n	BW	HW	HW/BW
**1 month**	Wt	5	16.2±2.2	91.2±13.7	5.65±0.57
	cKO	6	15.1±2.1	101.4±12.9	6.83±1.17
**3 months**	Wt	6	25.3±4.8	115.6±20.6	4.58±0.10
	cKO	7	23.1±4.2	115.3±24.4	4.99±0.39*
**TAC**	Wt, sham	5	26.6±5.1	122.7±21.3	4.63±0.15
	Wt, TAC	8	21.9±3.6	174.8±36.3*	7.96±0.87*
	cKO, sham	7	26.4±5.8	158.9±28.6	6.07±0.62
	cKO, TAC	9	21.3±2.9	224.4±50.6*†	10.57±1.98*†

Table S2. Overview of morphometric measurements. Abbreviations: n, number; BW, body weight (g); HW, heart weight (mg); HW/BW, heart weight to body weight ratio (mg/g); wt, wild type; cKO, cardiac specific Rbfox1 knock-out; TAC, transverse aortic constriction. *p<0.05 vs Wt or sham. †p<0.05 vs Wt, TAC
